# Uptake of pediatric patient-reported outcome and experience measures and challenges associated with their implementation in Alberta: a mixed-methods study

**DOI:** 10.1186/s12887-023-04169-w

**Published:** 2023-07-18

**Authors:** Sumedh Bele, Sarah Rabi, Muning Zhang, Sadia Ahmed, Elizabeth Oddone Paolucci, David W. Johnson, Hude Quan, Maria J. Santana

**Affiliations:** 1 Department of Pediatrics, Cumming School of Medicine, University of Calgary, 2500 University Dr NW, Calgary, AB T2N 1N4 Canada; 2 Department of Community Health Sciences, Cumming School of Medicine, University of Calgary, Calgary, Canada; 3 Patient Engagement Team, Alberta SPOR Support Unit, Calgary, Canada; 4 Cumming School of Medicine, Bachelor of Health Sciences Program, University of Calgary, Calgary, Canada; 5 Department of Surgery, Cumming School of Medicine, University of Calgary, Calgary, Canada; 6 Newborn, Child and Youth Strategic Clinical Network, Alberta Health Services Maternal, AB Calgary, Canada; 7 Department of Physiology and Pharmacology, Cumming School of Medicine, University of Calgary, Calgary, Canada; 8 Knowledge Translation Team, Alberta SPOR Support Unit, Calgary, Canada; 9 Data and Services Team, Alberta SPOR Support Unit, Calgary, Canada

**Keywords:** Patient-reported Outcome Measures (PROMs), Patient-reported Experience Measures (PREMs), Pediatrics, Mixed-methods study

## Abstract

**Background:**

Implementing Patient-reported Outcome Measures (PROMs) and Patient-reported Experience Measures (PREMs) is an effective way to deliver patient- and family-centered care (PFCC). Although Alberta Health Services (AHS) is Canada's largest and fully integrated health system, PROMs and PREMs are yet to be routinely integrated into the pediatric healthcare system. This study addresses this gap by investigating the current uptake, barriers, and enablers for integrating PROMs and PREMs in Alberta's pediatric healthcare system.

**Methods:**

Pediatric clinicians and academic researchers with experience using PROMs and PREMs were invited to complete a quantitative survey. Additionally, key stakeholders were qualitatively interviewed to understand current challenges in implementing pediatric PROMs and PREMs within AHS. Quantitative data gathered from 22 participants were descriptively analyzed, and qualitative data from 14 participants were thematically analyzed.

**Results:**

Participants identified 33 PROMs and 6 PREMs showing diversity in the types of pediatric PROMs and PREMs currently being used in Alberta and their mode of administration. The qualitatively identified challenges were associated with patients, family caregivers, and clinicians. The absence of system-level support, such as integration within electronic medical records, is considered a significant system-level challenge.

**Conclusions:**

The significant variation in the types of PROMs and PREMs used, the rationale for their use, and their mode of administration demonstrate the diverse and sporadic use of these measures in Alberta. These findings highlight the need for province-wide uniform implementation of pediatric PROMs and PREMs in Alberta. Our results could benefit healthcare organizations in developing evidence-based PROM and PREM implementation strategies in pediatrics.

**Supplementary Information:**

The online version contains supplementary material available at 10.1186/s12887-023-04169-w.

## Background

In recent years, there has been a shift in healthcare provision, pivoting towards a more Patient- and Family-Centered Care (PFCC) framework for healthcare decision-making [1, 2]. In pediatrics, PFCC emphasizes partnership and collaboration with patients and families when formulating and individualizing their treatment plans. The importance of such care strategies has led to the recognition of PFCC as a central indicator for high-quality health care in patient-clinician interactions [2, 3]. The goal of PFCC is to empower patients and their families in their care by ensuring that their voices are heard and respected. Instead of traditional physician-dominated consultations, patients are encouraged to participate in a dialogue surrounding their own healthcare decisions and develop a collaborative relationship with clinicians and health systems [4]. In recognizing what is important to patients, healthcare providers and health systems can adapt and improve their services to best-fit patients' and families' needs, a crucial step towards providing more comprehensive and efficacious healthcare [5].

One effective way to involve patients and families in conversations about their health is through the use of Patient-Reported Outcome Measures (PROMs) and Patient-Reported Experience Measures (PREMs) [6, 7]. PROMs and PREMs are standardized and validated questionnaires that allow patients to self-report their current health status and experiences receiving care, respectively [8]. PROMs inquire about a patient's functional capacity (generic or disease-specific) and wellbeing. They measure intrinsic outcomes, such as functional status and health-related quality of life (HRQOL) [8]. Disease-specific PROMs can help address particular disease symptoms impacting health conditions and outcomes [8]. Alternatively, PREMs measure care aspects related to the experience of a health encounter which includes patient-provider communication, the clinical environment, or efficiency in healthcare delivery. Thus, PREMs help capture patients' and families' feedback regarding their experience interacting with the healthcare system [8]. PREMs typically provide information for quality improvement or program evaluation initiatives. Together, results from PROMs and PREMs can be used to provide PFCC [7, 9].

Despite the indisputable benefits of using PROMs and PREMs to deliver PFCC, their implementation lags in routine pediatric clinical care [10]. Previous research has identified implementation barriers in adult patients, including the assurance of patient comprehension, fears of workflow obstruction, limited capacity to integrate responses into clinical care, and insufficient technological infrastructure to facilitate survey completion [11, 12]. The use of PROMs and PREMs in pediatric populations poses additional challenges, such as assessing the capacity of the patient to effectively comprehend survey questions and weighing the benefits of *by-proxy*survey completion, while still ensuring that the patient's voice is being heard [13].

In Canada, Alberta Health Services (AHS) provides all healthcare services within the province. AHS has established a *Patient First Strategy*, an organization-wide initiative to improve PFCC practices, including patient engagement and partnership [14, 15]. Within AHS, there are sporadic uses of PROMs and PREMs in clinical care and research, as well as a general lack of integration of PROMs and PREMs in routine clinical care, especially in pediatric health services. To facilitate province-wide implementation of pediatric PROMs and PREMs, it is essential first to understand the current use of these measures in Alberta. It is equally essential to explore the perspectives of current pediatric PROM and PREM users to understand current practices and the system-level challenges these users face. Therefore, this mixed-methods study aims to understand the current uptake of pediatric PROMs and PREMs in Alberta and the challenges associated with their implementation in routine clinical care.

## Methods

### Design

We conducted a convergent-parallel mixed-methods study comprised of quantitative and qualitative arms. The convergent-parallel study design is an approach of concurrently collecting complementary qualitative and quantitative data on the same phenomenon, followed by the convergence of data to facilitate a more comprehensive interpretation [16]. At the methods level, the integration of quantitative and qualitative data was achieved by bringing together data from two arms of analysis and comparison to understand the current uptake of pediatric PROMs and PREMs, as well as study participants' perceptions of the challenges associated with their implementation in Alberta.

### Ethics

Ethical approval for this study was obtained from the University of Calgary's Research Ethics Board (REB21-01441), with all study participants providing verbal consent prior to participating in the qualitative interview and implied consent prior to completing quantitative surveys. Administrative approval was also obtained from Alberta Health Services (AHS).

### Study setting and participants

This study was conducted in the Canadian province of Alberta. Alberta is Canada's fourth-most populous province and is served by AHS, Canada's first and largest province-wide fully integrated health system. The pediatric health ecosystem in Alberta includes two tertiary pediatric hospitals, Stollery Children's Hospital in Edmonton and Alberta Children's Hospital in Calgary. There are also five regional hospitals with a limited number of dedicated pediatric units. AHS has also established the Maternal Newborn Child and Youth Strategic Clinical Network (MNCY SCN™), one of 11 SCNs™ established as learning health systems to facilitate the translation of the latest evidence into practice. Additionally, the Alberta Children's Hospital Research Institute (ACHRI), affiliated with the University of Calgary, and the Women and Children's Health Research Institute (WCHRI), affiliated with the University of Alberta, serve as two major academic pediatric research institutions.

Participants of this study were healthcare professionals with experience using pediatric PROMs and PREMs and/or interested in using these measures in practice, quality improvement or for clinical research. Participants were comprised of pediatric clinicians, pediatric health services researchers and community care providers.

### Materials

For the study's quantitative arm, a survey was developed by our team to capture the current uptake of pediatric PROMs and PREMs in Alberta. This survey included 24 questions (see Additional file 1: Appendix 1). It focused on variables of interest, such as the name of the specific measure used, the type of health setting, mode of administering the measure, reasons for use (i.e., research, quality improvement, program evaluation, mental health, etc.), date of initial use, and methods of data reporting. This survey was designed in Qualtrics (Qualtrics, Provo, Utah, USA). For the qualitative arm of the study, an interview guide (see Additional file 1: Appendix 2) was developed to explore participants' knowledge, experiences, and perceptions of using pediatric PROMs and PREMs in their respective clinical practice or health services research projects.

### Data collection

All the data were collected between May 2021 and April 2022. Participants were recruited by disseminating study information in regular newsletters sent by the Departments of Pediatrics, ACHRI, WCHRI, and the AHS MNCY SCN™. The study invitation included a link to complete the anonymous survey through Qualtrics. In addition, a list of potential participants was compiled based on publicly available information about professions and positions in AHS. These potential participants were also sent emails inviting them to complete the survey. The study recruitment information shared through these channels also included an invitation to contact the study coordinator (SB) if the participants wished to be interviewed for the qualitative arm of the study. In addition, a snowball sampling approach was also utilized to recruit participants for the qualitative interviews.

All qualitative interviews were conducted virtually via Zoom. Before each interview, verbal consent was obtained from each participant. Interview participants received a $20 gift card to acknowledge their time and insights. All the interviews were audio-recorded and transcribed verbatim.

### Data analysis

Quantitative data collected through surveys were imported into MS Excel for descriptive statistical analysis. The users of pediatric PROMs and PREMs were categorized by their primary affiliation, clinical area of use, and whether they used only PROMs, PREMs, or both. A list of PROMs and PREMs was also compiled based on the responses provided in the quantitative survey. Lastly, the uses of pediatric PROMs were categorized into clinical care, research, and care evaluation. Similarly, the uses of pediatric PREMs were categorized into quality improvement, research, and care evaluation. A pie chart was created to demonstrate the frequency of different pediatric PROM and PREM modes of administration, which included via mail, phone, email, e-survey at the clinic, and paper (in the clinic or a secure portal).

Qualitative data were transcribed verbatim and imported into NVivo 12 (QRS International Pvt. Ltd Melbourne, Australia) to guide coding, organizing, and synthesis of the data. In the first step, two randomly chosen interview transcripts were coded independently by three research team members (SB, SR, and MZ) to develop a codebook, consistent of code definitions and associated quotes. Some changes were made to the codebook when additional categories were identified in subsequent interviews. Then, a researcher (SB) iteratively coded the remaining transcripts using this codebook and identified the patterns in the form of themes. Key statements demonstrating the beliefs of participants were attributed to themes and sub-themes. Final themes and sub-themes were shared with other team members to seek their feedback on thematic groupings and the selection of supporting quotes. These themes and sub-themes were then narratively described along with the de-identified quotes illustrating participants' core beliefs on the specific theme. Finally, results from quantitative and qualitative analyses were integrated and narratively interpreted to find convergence, divergence, contradictions, or relationships between quantitative and qualitative study findings.

## Results

The quantitative and qualitative data were collected concurrently, and a merging approach was used to integrate the findings from both arms of the study [17]. First, the findings from the quantitative arm of the study are reported, followed by the findings from the qualitative arm. Finally, qualitative and quantitative data integration was accomplished through a joint display and contiguous narrative approach at the interpretation and reporting level [17].

### Quantitative data

Twenty-eight people participated in the quantitative survey, however, six of these participants opened but did not complete any of the survey questions. Therefore, only data from 22 participants were included in the final quantitative analysis (See Table 1). Fifty-nine percent (*n* = 13) of the participants had a primary affiliation with AHS. The most common area where pediatric PROMs and PREMs were used was in general child health (18%, *n* = 4), followed by respirology (15%, *n* = 3) and rehabilitation (15%, *n* = 3). Most participants (60%, *n* = 13) completing the quantitative survey used both PROMs and PREMs.

**Table 1:** Study participants in quantitative arm and their use of pediatric PROMs and PREMs in Alberta

Characteristics	Category	Users (*n* = 22)
**Affiliation** **n (%)**	Alberta Health Services	13 (59%)
	University of Alberta	6 (27%)
	University of Calgary	3 (14%)
**Area of use** **n (%)**	General Child Health	4 (18%)
	Respirology	3 (15%)
	Rehabilitation	3 (15%)
	Rheumatology	2 (10%)
	Mental Health	2 (10%)
	Oncology	1 (4%)
	Neurology	1 (4%)
	Critical Care	1 (4%)
	Dermatology	1 (4%)
	Speech-Language Pathology	1 (4%)
	Nutrition Services	1 (4%)
	Neonatology	1 (4%)
	Hematology	1 (4%)
**Use of PROMs and PREMs** **n (%)**	Both PROMs and PREMs	13 (60%)
	Only PROMs	8 (36%)
	Only PREMs	1 (4%)

**Fig. 1:**
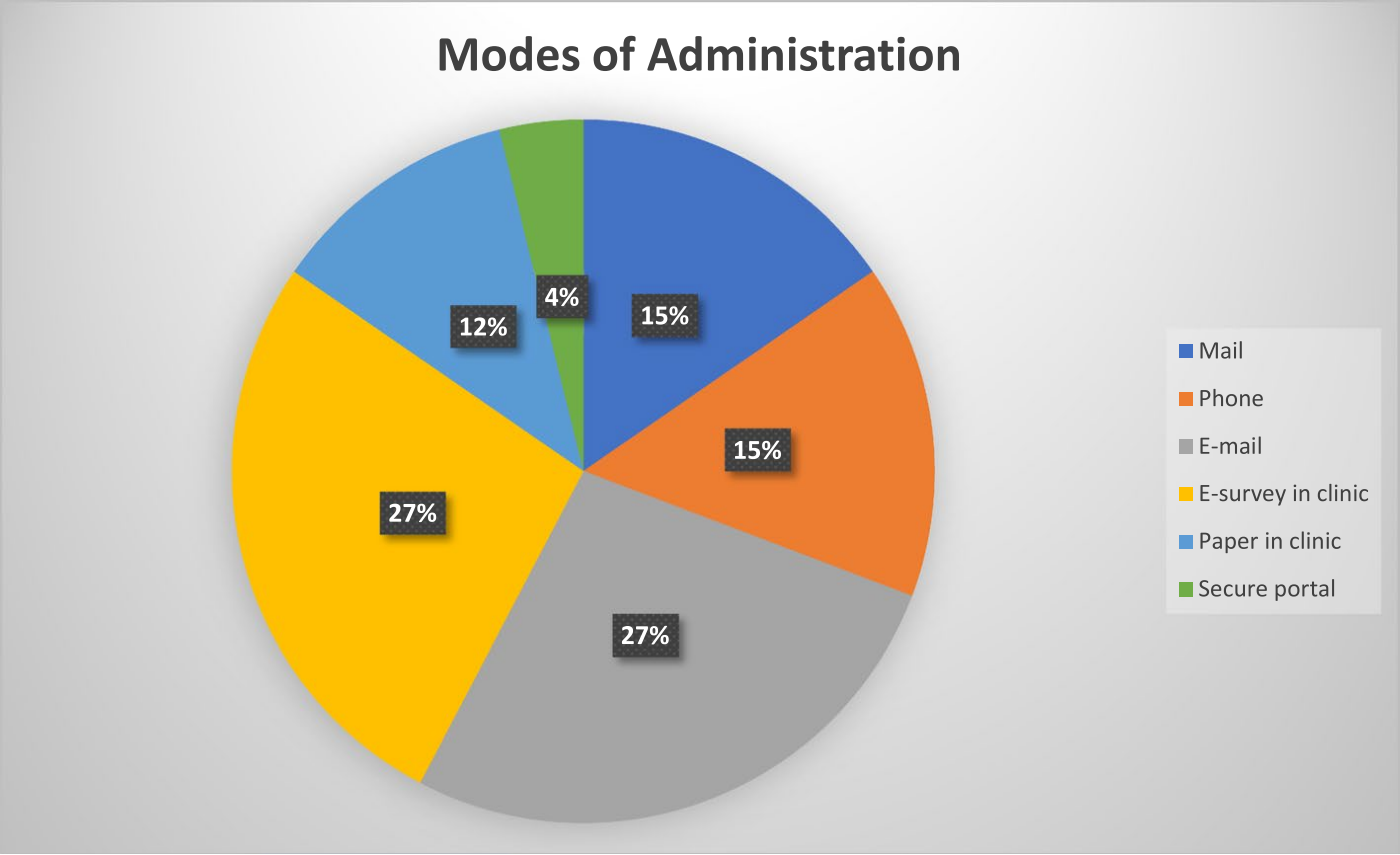
Mode of administration for PROMs and PREMs

One participant did not provide information on their uses for pediatric PROMs, so among 21 respondents (See Table 2), the most common reason for PROM use was research (81%, *n* = 17), followed by clinical care (71%, *n* = 15) and care evaluation (52%, *n* = 11). Only 14 participants used PREMs, among which the most common application was for research (71%, *n* = 10), followed by quality improvement (64%, *n* = 9) and care evaluation (57%, *n* = 8). Since participants may have been using pediatric PROMs and PREMs for multiple purposes, the numbers reported for the types of uses were not mutually exclusive. The most common modes of administering PROMs and PREMs were through email (27%, *n* = 7) and electronic completion at the healthcare facility (27%, *n* = 7) (See Fig. 1).

**Table 2:** Types of uses for pediatric PROMs and PREMs in Alberta

Types of measures	Type of use	Uses (n, %)
**PROMs (*n* = 21)**	Research	17 (81%)
	Clinical Care	15 (71%)
	Care Evaluation	11 (52%)
**PREMs (*n* = 14)**	Research	10 (71%)
	Quality Improvement	9 (64%)
	Care Evaluation	8 (57%)

There was significant variation in the current use of pediatric PROMs and PREMs in Alberta (See Table 3). In total, 33 unique PROMs were identified by the participants. The pediatric PROMs used in Alberta ranged from generic instruments such as the EQ-5D-Youth [18] and Pediatric Quality of Life Inventory (PedsQL™) [19], to disease-specific measures like the Knee injury and Osteoarthritis Outcome Score (KOOS) [20] and Children’s Dermatology Life Quality Index (CDLQI) [21]. On the other hand, only six unique pediatric PREMs were identified across all participants. The pediatric PREMs identified included generic PREMs, such as the Child Hospital Consumer Assessment of Healthcare Providers and Systems (Child-HCAHPS) [22], and condition-specific PREMs like the Measure of Processes of Care (MPOC) [23].

**Table 3:** Pediatric PROMs and PREMs currently being used in Alberta

**Type of measure**	**Name of the measure**
PROMs	1. Pediatric Quality of Life Inventory (PedsQL) 2. Childhood Health Assessment Questionnaire (CHAQ) 3. Patient-Reported Outcomes Measurement Information System (PROMIS) 4. EQ -5D—Youth 5. Pain Catastrophizing Scale – Child Version (PCS-C) 6. Pediatric- Migraine Disability Assessment Scale (PedMIDAS) 7. Children's Dermatology Life Quality Index (CDLQI) 8. State-Trait Anxiety Inventory (STAI) 9. Parenting Stress Index (PSI) 10. Parental Self Efficacy Scale (BPSES) 11. Breastfeeding Self-Efficacy Scale (BSES) 12. Peak Pruritus Numerical Rating Scale (Pruritus-NRS) 13. Patient Oriented Scoring for Atopic Dermatitis (PO-SCORAD) 14. Knee injury and Osteoarthritis Outcome Score (KOOS) 15. Tampa Scale for Kinesiophobia (TSK) 16. Anterior Cruciate Ligament-Return to Sport after Injury index (ACL-RSI) 17. Anterior Cruciate Ligament Quality of Life (ACL QOL) 18. Patient Specific Functional Scale (PSFS) 19. KIDSCREEN 20. General Anxiety Disorder-7 (GAD-7) 21. Child Behaviour Checklist (CBCL) 22. The Behavior Assessment System for Children (BASC -3) 23. Children's Communication Checklist- 2 (CCC-2) 24. SNAP-IV Teacher and Parent Rating Scale 25. Conners 3rd Edition (Conners 3) 26. Social Responsive Scale (SRS -2) 27. Attention-deficit/hyperactivity disorder (ADHD)-IV Rating Scale 28. Developmental Coordination Disorder Questionnaire (DCDQ) 29. Parent Perception of Uncertainty Scale (PPUS) 30. American College of Rheumatology Response Measure (ACR20) 31. Bayley Scales of Infant and Toddler Development 32. Behavior Rating Inventory of Executive Function (BRIEF) Questionnaire 33. OSA-18 Quality of Life Survey
PREMs	1. Child Hospital Consumer Assessment of Healthcare Providers and Systems (Child-HCAHPS) 2. Measure of Processes of Care (MPOC) 3. Pediatric Trust in Physician Scale (Pedi-TiPS) 4. Parent Satisfaction Questionnaire (PSQ) 5. Assessment of Registered Dietitian Care Survey (ARCS) 6. Alberta Family Integrated Care Parent Experiences Survey

Overall, participants identified 33 PROMs and 6 PREMs showing diversity in the types of pediatric PROMs and PREMs currently being used in Alberta, with their mode of administration ranging from emails to traditional paper–pencil modes. The purpose of using PROMs and PREMs were similarly diverse, including research, clinical care, quality improvement, and care evaluation.

### Qualitative data

We interviewed 14 participants for the qualitative arm of this study, with thematic successfully reached (see Table 4). While nine of the 14 participants openly expressed interest in being interviewed; all of them willingly consented. Two participants were purposively recruited because they were known users of pediatric PROMs and PREMs. In addition, two participants were included through snowball sampling and one participant was reached out to using their publicly available profile. Half of the participants were primarily affiliated with AHS, with the remaining at the University of Alberta, University of Calgary, or a community organization.

**Table 4:** Characteristics of study participants in the qualitative arm of the study (*n* = 14)

Characteristics	Category	Participants (*n* = 14)
Sex n (%)	Male	3 (21%)
	Female	11 (79%)
Primary affiliation n (%)	Alberta Health Services	7 (50%)
	University of Calgary	4 (29%)
	University of Alberta	2 (14%)
	Community Organization	1 (7%)

All interviews were held over a period of nine months (from May 2021 to January 2022) and lasted between 29 to 48 min in length. Table 5 shows themes and sub-themes around the current use of pediatric PROMs and PREMs in Alberta, as well as the challenges associated with their implementation in routine clinical care. Qualitative interviews were conducted by SB and SA, who have received graduate-level academic training in qualitative research methodology and have experience conducting interviews and focus groups. Below we have described the themes and sub-themes surrounding the current use of pediatric PROMs and PREMs in Alberta, as well as the challenges associated with their implementation in routine clinical care.

**Table 5:** Themes and sub-themes identifying current use, and challenges in using pediatric PROMs and PREMs in Alberta

Themes	Subthemes	Example Quotes
**1. Use of Pediatric PROMs and PREMs in Alberta**	***a. Specialty-specific implementation***	*“I think most of everything we measure in pain medicine, including pain severity, is patient-reported”* (HCP – 02) *“Our other study is a care for disease study for children who have neurodevelopmental disabilities.*” (HCP -10)
	***b. Rationale***	
	i. Offering greater insights into patient’s conditions	*“I think PROMs help with getting a better view, how the patient feels overall, so I think that is where I'm confident that it really helps us.”* (HCP—02)
	ii. Tracking outcomes over time	*“The next three months we're going to do again and if the next time we have the same we’re- we’re going to make this step that we're going to increase treatment or stop treatment.”* (HCP – 11)
	iii. Promoting shared decision making	*“I think it helps really a lot with decision making and trying to make these difficult decisions of stopping or adding a medication.”* (HCP – 04)
	iv. Facilitating patient management	*“I think they're very important to integrate a patient's perspective, it helps you to make a good management plan going forward and you've got often, you get what matters to the patient rather than what you think matters to the patient.”* (HCP – 10)
	***c. Training requirements***	*“As a clinician, one needs to be familiar with the specific tools and how they’re used, what they show, broadly, what's the evidence behind them? Because I think it's important to understand that broadly…”*(HCP—06) *“They need those evidence-informed teaching tools to be able to provide that consistent information to families that will make them much happier because they won't be confused and frustrated. And that's a better experience (HCP – 05)*
**2. Administration of PROMs and PREMs**	***a. Modality***	*“For us they are all on paper and then we have to transfer them into the electronic system, which also brings another complication because that could potentially also again, like you know, put a bias in it because we don't transfer exactly what has been put on paper”* (HCP -03) *“All the PREMs and PROMs will be under that sink (if in paper form), in the cupboard under the sink. It's much easier with electronic data collection platforms now”* (HCP—01)
**4. Challenges Associated with PROMs and PREMs implementation**	***a. Clinician-associated challenges***	
	i. Limited capacity to address PROMs and PREMs identified issues	*“You know, you're asking a patient ‘tell me how you feel?’ and then they tell you ‘I feel crap,’ and then you’re saying, ‘I'm sorry, we don't have the resources to do anything about it.’ Right?”* (HCP – 03)
	ii. Personal apprehension about the use of PROMs and PREMs	*“I suspect some will intuitively get it more readily than others. Some will be a bit slower; some will say yeah, this is useless.”* (HCP – 01)
	iii. Other barriers	*“I think you need to know the limitations of the PROMs and PREMs, and you also need to know if they fit in the- in the context.”* (HCP – 12)
	***b. Patient and family-associated challenges***	
	i. Lack of understanding of the importance of PROMs and PREMs	*“What is my most experience is that you make sure that patients understand what patient-reported outcome means.”* (HCP -11)
	ii. Capacity to complete PROMs and PREMs	*“… those are usually much more extensive PROMs, which sometimes is a bit of a burden on the families, of course, because it's a lot of questionnaires that need to be filled.”* (HCP – 14)
	***c. System-level challenges***	
	i. Connect Care	*“Connect Care will help us with that, we're not there yet, we're working on it.”* (HCP – 03)
	ii. Policy-mandate	*“If you look at cancer care, I mean they've got this PREMs and PROMs stuff down because they've had bundles of money for years because Cancer Research is actually embedded in the act, in the Cancer Care Act. Did you know that? Do you know that that's not embedded in any other clinical care? But it's embedded in the Cancer Care Act, which is why if you've got a policy, the money has to follow the policy”* (HCP – 01)
	iii. Impact of Covid-19 pandemic	*“It's very hard to do PROMs because they're on paper (and appointments are) through Zoom, so we miss a lot of PROMS and PREMs”* (HCP – 12)

### Use of Pediatric PROMs and PREMs in Alberta

One purpose of the qualitative inquiry was to understand how PROMs and PREMs are being used in pediatric health settings across Alberta. This larger theme focused on the speciality-specific implementation and participant rationales behind PROM and PREM use.

### Specialty-specific implementation

Since study participants came from diverse backgrounds, they were able to provide an overview of the different clinical areas in which PROMs and PREMs are used.

“Our other study is a care for disease study for children who have neurodevelopmental disabilities.” (HCP -10).

“I think most of everything we measure in pain medicine, including pain severity, is patient-reported” (HCP – 02).

Often different health systems will choose a few generic or disease specific PROMs and PREMs to implement in routine clinical care, however, these statements demonstrate how the study participants also came from diverse clinical backgrounds (i.e., pain medicine) where great importance is paid to the use of patient-reported measures.

### Rationale for using PROMs and PREMs

Despite a lack of province-wide implementation of pediatric PROMs and PREMs, some clinicians and health service researchers were using PROMs and PREMs. After probing these participants further on their rationale for using these measures, four additional sub-themes were revealed about their beliefs about the utility of these measures.

### Offering greater insights into patient’s conditions

Study participants considered these measures as tools that provide them more information about how a certain disease or health encounter impacts their patients (and families).

“I think PROMs help with getting a better view, how the patient feels overall, so I think that is where I'm confident that it really helps us.” (HCP—02).

### Tracking outcomes over time

Participants also endorsed the use of PROMs and PREMs to track patients' trajectories by monitoring patients' health outcomes and experiences over more extended periods of time. They believed that such long-term monitoring of PROMs and PREMs data in clinical care helps them and their clinical teams to improve their patients’ health outcomes and experiences.

“The next three months we're going to do again and if the next time we have the same we’re- we’re going to make this step that we're going to increase treatment or stop treatment.” (HCP – 11).

### Promoting shared decision making

Participants believed that using PROMs and PREMs helps to promote shared-decision making. According to them, since these measures are directly reported by patients and/or their family caregivers, they can help evaluate different treatment options that matter most to the patients and/or family caregivers.

“I think it helps really a lot with decision making and trying to make these difficult decisions of stopping or adding a medication.” (HCP – 04).

### Facilitating patient management

Participants at the frontlines of providing clinical care felt that PROMs and PREMs offered them greater insights into patients' conditions, highlighting that the patient perspectives that were captured by PROMs and/or PREMs enabled them to better manage their patients’ symptoms and provide PFCC.

“I think they're very important to integrate a patient's perspective, it helps you to make a good management plan going forward and you've got often, you get what matters to the patient rather than what you think matters to the patient.” (HCP – 10).

### Training requirements

Although participants either knew about PROMs and PREMs, or were already using them in their clinical practice or health services research, they highlighted the desire to receive more training on the science behind developing PROMs and PREMs, and the optimal ways to use PROMs and PREMs data in clinical care.

“As a clinician, one needs to be familiar with the specific tools and how they’re used, what they show, broadly, what's the evidence behind them? Because I think it's important to understand that broadly…”(HCP—06).

“They need those evidence-informed teaching tools to be able to provide that consistent information to families that will make them much happier because they won't be confused and frustrated. And that's a better experience (HCP – 05).

### Administration of pediatric PROMs and PREMs

Our quantitative data showed that participants were using different modes to administer and collect PROMs and PREMs data, so we explicitly asked participants about their experience using different modes of administration and any specific challenges associated with them. All the qualitative data on administration modality for PROMs and PREMs were grouped into this theme.

“For us they are all on paper and then we have to transfer them into the electronic system, which also brings another complication because that could potentially also again, like you know, put a bias in it because we don't transfer exactly what has been put on paper” (HCP -03).

“All the PREMs and PROMs will be under that sink (if in paper form), in the cupboard under the sink. It's much easier with electronic data collection platforms now” (HCP—01)

In this theme, participants highlighted the challenges with traditional modes of administering PROMs and PREMs (i.e., paper-based) and underscored the importance of moving towards electronic administration. Study participants also proposed creating a repository of PROMs and PREMs results, which could be utilized for multiple purposes, including clinical care and research.

### Challenges associated with PROM and PREM implementation

Study participants faced, or anticipated facing, several challenges with implementing PROMs and PREMs in clinical care. The majority of the challenges shared by study participants were either associated with the clinicians, patient and family members, or health system at large, therefore, we divided this theme into these three sub-themes, respectively.

### Clinician-associated challenges

#### Limited capacity to address PROM and PREM identified issues

Although participants overwhelmingly supported the use of PROMs and PREMs in Alberta, they were also sceptical about their abilities to address some of the issues identified by the measures, suggesting that they might be outside their scope of practice. Participants stated that sometimes their patients might disclose information about how their clinical condition might has impacted the patients’ social life or mental health, but healthcare providers might not be trained to deal with such issues or do not have adequate supports.

“You know, you're asking a patient ‘tell me how you feel?’ and then they tell you ‘I feel crap,’ and then you’re saying, ‘I'm sorry, we don't have the resources to do anything about it.’ Right?” (HCP – 03).

#### Personal apprehension about the use of PROMs and PREMs

According to study participants, some of their peers might have personal apprehensions about the utility of PROMs and PREMs, as well as the non-suitability of specific PROMs and PREMs to clinicians' personal style of practicing medicine. The use of PROMs and PREMs was compared to any other intervention, which have early adopters and laggards, who are slow to adopt to the change. Such apprehension may be due to various reasons including personal apprehensions about the intervention. Participants believed that one of the reasons for slow adoption might be that some of their colleagues consider PROMs and PREMs to be a nuisance rather than a useful tool for clinical practice.

“I suspect some will intuitively get it more readily than others. Some will be a bit slower; some will say yeah, this is useless.” (HCP – 01)

#### Other barriers

Some other barriers mentioned by the participants included interruptions in clinical flows and an inability to select the right measure for the right scenario. One important barrier that is relevant to the implementation of PROMs and PREMs in Alberta was identified as “implementation context”. Participants felt that because of the size of Alberta’s health system, each pediatric healthcare facility had cultivated a distinct work culture. Therefore, a lack of understanding about each clinical context could constitute a major barrier to the province-wide implementation of PROMs and PREMs.

“I think you need to know the limitations of the PROMs and PREMs, and you also need to know if they fit in the- in the context.” (HCP – 12)

#### Patient and family-associated challenges

Study participants believed that it is equally essential to engage patients and family caregivers in order to successfully use PROMs and PREMs in routine clinical care.

#### Lack of understanding of the importance of PROMs and PREMs

Since PROMs and PREMs require active engagement from patients and family members, participants underlined the importance of ensuring the patients and families understand the value of using such measures.

“What is my most experience is that you make sure that patients understand what patient-reported outcome means.” (HCP -11).

#### Capacity to complete PROMs and PREMs

Similarly, participants felt that some of the patients and family members might be interested in completing these measures, however, they might not have the capacity to actually complete them. This lack of capacity could be attributed to issues such as, parents’ burden to care for more than one child, the length of PROM and PREM measures, and a lack of language proficiency. Therefore, limited capacity to complete these measures, was viewed as a barrier that could potentially hamper the uptake of PROMs and PREMs in routine clinical care.

“… those are usually much more extensive PROMs, which sometimes is a bit of a burden on the families, of course, because it's a lot of questionnaires that need to be filled.” (HCP – 14).

#### System-level challenges

Lastly, several challenges were associated with the infrastructure and policies within AHS. Some of the system-level changes were also attributed to the COVID-19 pandemic.

#### Connect care

AHS has recently rolled out a province-wide electronic medical record system called Connect Care, with most participants expressing very high hopes for Connect Care's ability to facilitate the use of pediatric PROMs and PREMs in Alberta. Currently, however, the lack of integration of PROMs and PREMs within Connect Care was identified as a major system-level challenge.

“Connect Care will help us with that, we're not there yet, we're working on it.” (HCP – 03)

#### Policy-mandate

Participants believed that without policy mandates to incorporate PROMs and PREMs in routine clinical care, it would be difficult to scale and spread the use of PROMs and PREMs in Alberta. A similar experience was shared by one key participant who explained the impact of policy making on increasing the uptake of PROMs and PREMs in cancer care.

“If you look at cancer care, I mean they've got this PREMs and PROMs stuff down because they've had bundles of money for years because Cancer Research is actually embedded in the act, in the Cancer Care Act. Did you know that? Do you know that that's not embedded in any other clinical care? But it's embedded in the Cancer Care Act, which is why if you've got a policy, the money has to follow the policy” (HCP – 01).

#### Impact of COVID-19 pandemic

Lastly, participants shared some challenges they faced in using PROMs and PREMs that were specific to the COVID-19 pandemic. At the time of data collection, AHS was using virtual tools to provide clinical care for non-acute patients. Several participants shared that they did not believe this format was not suitable for administering PROMs or PREMs.

“It's very hard to do PROMs because they're on paper (and appointments are) through Zoom, so we miss a lot of PROMS and PREMs” (HCP – 12)

#### Convergence of quantitative and qualitative findings

Table 6 is a joint display illustrating the convergence of our findings from the quantitative and qualitative arms. As is evident in the findings from both arms of the study, most quantitative and qualitative findings complemented each other. In Table 6, the left column lists major findings from the quantitative arm, and the right column highlights complementary findings captured through qualitative interviews. The quantitative and qualitative findings were predominantly convergent. We did not find any divergent or contradictory findings. The qualitative arm of the study was more exploratory, so it provided additional unique findings the highlighted some of the challenges associated with implementing pediatric PROMs and PREMs in Alberta.

**Table 6:** Joint display illustrating the convergence of findings from qualitative and quantitative arms of the study

QUANTITATIVE FINDINGS (survey results)	QUALITATIVE FINDINGS ( sample quotes from interviews)
**Diversity in affiliation and specialty among users of pediatric PROMs and PREMs** • More than half of the participants are primarily affiliated with AHS but many of them are also clinical or health services researchers • Use of pediatric PROMs and PREMs reported from 13 pediatric specialties	**Specialty-specific implementation of pediatric PROMs and PREMs** • *“So my portfolio is half research and half clinical work”(HCP 03)* • *“I do some general neurology as well for call and service and then I spend the other half of my time in clinical research*”(HCP 08) • *“I'm a pediatric rheumatologist so we see patients with arthritis* (HCP 06) • *“We’re specifically looking at children with medical complexity”(*HCP 03)
**Type of uses for pediatric PROMs and PREMs** • Uses for pediatric PROMs and PREMs include research, clinical care, care evaluation, and quality improvement	**The rationale for using pediatric PROMs and PREMs** • *“I think that would be the ultimate, is having a model of care where each individual patient prioritizes their own PROMs, and we can look at those outcomes over time”* (HCP 14) • *“The other thing in terms of experience is when, when you, when providers have a good relationship with parents, that will spin off to good unit, better unit culture”*(HCP 10) • *“Our goal is to, to take this evidence to Alberta health services and say, Hey, you are doing it for evaluation purpose”* (HCP 13)
**Variety in pediatric PROMs and PREMs currently used in Alberta** • Participants identified 33 unique PROMs and six unique PREMs	**Pediatric PROMs and PREMs mentioned during the interviews** • *“I have used the PedsQL for health-related quality of life”* (HCP 10) • *“we used like Childhood Health Assessment Questionnaire (CHAQ)”* (HCP 09) • *“REEL questionnaire, again, which is the speech delay or speech questionnaire”* (HCP 07)
**Variation in the mode of administering pediatric PROMs and PREMs** • The participant reported modes of administration included emails, mail, phone, electronic survey, and on-paper at the clinic	**Challenges associated with the modality of administering pediatric PROMs and PREMs** • *“the Secretary mails out that Bailey three questionnaire and they the parents are asked to fill it in and bring it with them to the clinic when they come”* (HCP 08) • *“For some of the studies we do have iPads and we try to let them do it on an iPad”* (HCP 02) • *“The care coordination study that I described we are actually doing it by phone”* (HCP 03) • *“we're probably going to do an email survey link”* (HCP 12)

## Discussion

The growing evidence-base around the effectiveness of PROMs and PREMs in supporting PFCC is irrefutable [8, 24]. AHS is Canada's largest integrated health system and has enacted the *Patient First Strategy *[25]. However, as shown by the results of our study, PROMs and PREMs are not consistently incorporated into routine pediatric clinical care. This mixed-methods study was conducted to understand the current use of pediatric PROMs and PREMs in Alberta and the challenges associated with their implementation in routine clinical care.

This study identified great variation in the types of health settings where pediatric PROMs and PREMs are currently being used. It also showed the diversity in the types of PROMs and PREMs and the purposes for using them. The modes of administering PROMs and PREMs ranged from the traditional paper–pencil mode to email and electronic platforms. Most of the study participants used PROMs and PREMs for research, followed by clinical care, quality improvement, and care evaluation. The challenges in implementing PROMs and PREMs in routine clinical care were associated with physicians, patients and family caregivers, and the overall health system. In Alberta, women account for over 80% of the healthcare workforce, which explains the proportionally higher number of women in our study.

A recent systematic review emphasized that organizations implementing PROMs need to invest time and resources into ‘designing’ a PROMs strategy and ‘preparing’ the organization to use PROMs [26]. Another recent study from the Netherlands found that PREMs implementation strategies need to focus on designing and preparing implementation at the patient-clinician interaction level [27]. These studies highlight healthcare organizations' role in facilitating the implementation of PROMs and PREMs.

The quantitative arm of this study found 33 PROMs and 6 PREMs currently being used in pediatric health systems in Alberta. This number might look large, but there are hundreds of PROMs and PREMs developed by researchers and health systems based on their specific needs [28–31]. Another recently published systematic review of childhood PROMs identified 89 generic PROMs, including 110 versions [32]. Therefore, the number of disease or condition-specific PROMs could be considerably greater. Similarly, our team's systematic review of pediatric PREMs identified 49 pediatric PREMs being used worldwide [29]. This illustrates that large health systems like AHS need to strike a balance between standardization by implementing a few PROMs and PREMs across the province and adaptation according to individual unit or clinician needs.

Participants offered several rationales for using PROMs and PREMs. According to them, these measures offered greater insights into patients’ conditions and experiences, promoted shared decision-making, facilitated patient management, and helped track patient outcomes and experiences over time. These were the primary rationales for developing PROMs and PREMs and have been reported widely in the adult population as well [8]. Our study confirms similar uses of these measures in pediatric healthcare. At a broader level, participant-identified uses of pediatric PROMs and PREMs included research, clinical care, quality improvement, and care evaluation. These uses are also highlighted in relevant published literature on this topic [8, 31]. In fact, the literature highlights the potential of PROMs in transforming healthcare if the individualized and aggregated PROMs data is used in clinical care, research, or care evaluation [33]. Similar use of PREMs also has great potential to improve health system performance [34]. The findings from our study show that clinicians and health service researchers in Alberta rightly use pediatric PROMs and PREMs but face many challenges identified through the qualitative arm of the study. Some of the challenges, such as personal apprehensions about PROMs and PREMs, and the inability to address issues identified by PROMs and PREMs, can be mitigated by engaging clinicians in the process of selecting these measures and jointly creating clinical management pathways [35, 36]. Patient and family-associated challenges could be mitigated by educating patients and families, and supporting them through the completion of PROMs and PREMs before and after clinical encounters [37].

The major system-level challenge identified in the literature and our study is the lack of integration of PROMs and PREMs within electronic medical records [11]. AHS is currently rolling out Connect Care, a province-wide electronic medical records system [38]. AHS plans to implement PROMs and PREMs through Connect Care in the future, so some of the system-level challenges may be mitigated. Another important challenge identified by participants was associated with policy mandates by health systems to integrate PROMs and PREMs in clinical care. The US Food & Drug Administration and the European Medicines Agency have mandated the use of PROMs to support labelling claims [39, 40]. Similarly, the National Health Services (NHS) of England has mandated the use of PROMs for certain elective surgeries [41]. These policy mandates have been effective in standardizing the use of PROMs across the healthcare system. Therefore, AHS should also consider developing recommendations and policy mandates to support the use of pediatric PROMs and PREMs across Alberta.

There is growing evidence around the use of pediatric PROMs and PREMs in different health systems worldwide [28, 29, 31]; however, according to our knowledge, this is the first study to use a mixed-methods approach to comprehensively understand the experiences and perspectives of PROMs and PREMs users within a large integrated health system. Some of the findings from this study would be helpful for other pediatric health systems, recognizing however that every health system is unique and some of our findings may be highly specific to the Alberta context. AHS could utilize the findings from this study to develop a province-wide pediatric PROMs and PREMs implementation strategy.

### Strengths and limitations

Combining qualitative and quantitative methods within the same study allows for a more comprehensive understanding of the phenomenon under investigation by strengthening and validating the results [42]. This study's convergent mixed methods approach gathered complementary data to provide a comprehensive and multidimensional understanding of the uptake of pediatric PROMs and PREMs in Alberta and the system-level challenges associated with their implementation. There were also several limitations of this study. First, despite our efforts to reach all the users of pediatric PROMs and PREMs for the quantitative arm of the study, we might have missed some of the users of pediatric PROMs and PREMs in Alberta. In addition, participation in this study was voluntary, so selection bias might have excluded participants who use PROMs and PREMs but do not wish to participate in this study. The qualitative arm of the study was more exploratory, so it elicited more unique findings. However, due to the concurrent nature of this study, unique themes identified through qualitative interviews could not be measured through quantitative surveys. Lastly, the COVID-19 pandemic had disrupted the healthcare system, so the results might not reflect post-pandemic times. Currently, a large research program is underway to generate evidence to support the province-wide integration of pediatric PROMs and PREMs in Alberta using KidsPRO, an innovative e-health solution. The KidsPRO program will utilize findings from this study. However, future studies should comprise a larger sample size and be conducted in non-pandemic times.

## Conclusion

Although integrating PROMs and PREMs in clinical care is recognized as an effective way to deliver PFCC, their use is limited in pediatrics healthcare systems in Alberta. This study shows the significant variation in the types of PROMs and PREMs, rationale for their use, and mode of administration to demonstrate the diverse and sporadic use of these measures in Alberta. Our study also highlights a lack of a standardized approach to implementing pediatric PROMs and PREMs in Alberta. The findings from this study could help healthcare organizations like AHS to develop evidence-based PROM and PREM implementation strategies in routine pediatric clinical care.

## Supplementary Information

**Additional file 1: Appendix 1. **Quantitative survey to collect data on the current use of pediatric PROMs and PREMs in Alberta. **Appendix 2.** The interview guide to collect qualitative data on the use of pediatric PROMs and PREMs in Albertaand the challenges associated with their implementation.

## Data Availability

The data that support the findings of this study are available from the corresponding author, MS, upon reasonable request.

## Abbreviations

ACHRI: Alberta Children’s Hospital Research Institute
ACL-RSI: Anterior Cruciate Ligament-Return to Sport after Injury index
ACL QOL: Anterior Cruciate Ligament Quality of Life
ADHD IV Rating Scale: Attention-deficit/hyperactivity disorder (ADHD)-IV Rating Scale
AHS: Alberta Health Services
ARC20: American College of Rheumatology Response Measure
ARCS: Assessment of Registered Dietitian Care Survey
BASC-3: Behavior Assessment System for Children—3
BPSES: Parental Self Efficacy Scale
BRIEF: Behavior Rating Inventory of Executive Function
BSES: Breastfeeding Self-Efficacy Scale
CBCL: Child Behaviour Checklist
CCC-2: Children's Communication Checklist- 2
CDLQI: Children's Dermatology Life Quality Index
Child-HCAHPS: Child Hospital Consumer Assessment of Healthcare Providers and Systems
CHAQ: Childhood Health Assessment Questionnaire
Conners 3: Conners 3rd Edition
DCDQ: Developmental Coordination Disorder Questionnaire
GAD 7: General Anxiety Disorder-7
HRQOL: Health-Related Quality of Life
KOOS: Knee injury and Osteoarthritis Outcome Score
MPOC: Measure of Processes of Care
MNCY SCN: Maternal Newborn Child and Youth Strategic Clinical Network
NHS: National Health Services
PCS-C: Pain Catastrophizing Scale – Child Version
PedMIDAS: Pediatric- Migraine Disability Assessment Scale
PedsQL: Pediatric Quality of Life Inventory
Pedi-TiPS: Pediatric Trust in Physician Scale
PFCC: Patient- and Family-Centered Care
PO-SCORAD: Patient Oriented Scoring for Atopic Dermatitis
PPUS: Parent Perception of Uncertainty Scale
PREMs: Patient-Reported Experience Measures
PROMIS: Patient-Reported Outcomes Measurement Information System
PROMs: Patient-Reported Outcome Measures
Pruritus – NRS: Peak Pruritus Numerical Rating Scale
PSI: Parenting Stress Index
PSFS: Patient Specific Functional Scale
PSQ: Parent Satisfaction Questionnaire
SRS -2: Social Responsive Scale
STAI: State-Trait Anxiety Inventory
TSK: Tampa Scale for Kinesiophobia
WCHRI: Women and Children's Health Research Institute
