# Supplementary material for: Uptake of pediatric patient-reported outcome and experience measures and challenges associated with their implementation in Alberta: a mixed-methods study
Source: BMC Pediatr. 2023 Jul 18;23:369. doi: 10.1186/s12887-023-04169-w (PMC10353095; doi:10.1186/s12887-023-04169-w)
Supplement: Supplementary file 1 — Additional file 1: Appendix 1. Quantitative survey to collect data on the current use of pediatric PROMs and PREMs in Alberta. Appendix 2. The interview guide to collect qualitative data on the use of pediatric PROMs and PREMs in Albertaand the challenges associated with their implementation. [file 12887_2023_4169_MOESM1_ESM.docx]

**APPENDIX 1**: Quantitative survey to collect data on the current use of pediatric PROMs and PREMs in Alberta

**Use of PROMS/PREMS in Pediatric Care Settings - Jan 2021**

**Start of Block: Default Question Block**

Q1 **Use of PROMs and PREMs in Albertan pediatric health systems** We would like to invite you to participate in a short survey as part of an environmental scan to understand the uptake of Patient-reported Outcome Measures (PROMs) and Patient-reported Experience Measures (PREMs) in Canadian pediatric healthcare systems. Patient-reported Outcome Measures (PROMs) are used to assess a patient's health status at a particular point in time (e.g. EQ-5D). Patient-reported Experience Measures (PREMs) are used to measure patient's perceptions of their experience while receiving care (e.g. HCAHPS). In pediatric settings, PREMs and PROMs may rely on reports from parents or guardians. This survey is being conducted for internal use; therefore, the results of this survey will only be used to inform our next steps to promote and support the integration of PROMs and PREMs within Canadian pediatric healthcare settings.

We are inviting you to complete this survey because you might be using PROMs and PREMs in your pediatric healthcare setting or be aware of their use in your organization. Please note that your participation is completely voluntary. Please let us know if you would like to receive the results by emailing: [s*****.***e@ucalgary.ca](mailto:sumedh.bele@ucalgary.ca)

This study has been approved by the University of Calgary's Conjoint Health Research Ethics Board (REB ID#21-0141 ). If you have any questions or concerns about the survey, you can contact the research coordinator at [sumedh.bele@ucalgary.ca](mailto:sumedh.bele@ucalgary.ca)

Q2 What is the name of your organization?

Q3 What is the area of your work/research?

Q4 What is your position within your organization?

Q5 Does your organization/clinic use Patient-reported Outcome Measures (PROMs)?

o Yes

o No

Q6 Does your organization/clinic use Patient-reported Experience Measures (PREMs)?

o Yes

o No

Q7 Which PREMS and/or PROMs do you use?

Q11 Do you or your team use PROMs as part of healthcare delivery?

o Yes

o No

Q12 Do you or your team uses PROMs in research?

o Yes

o No

Q14 Do your or your team use PROMs for program evaluation?

o Yes

o No

Q15 Do you or your team use PREMs in research?

o Yes

o No

Q16 Do you or your team use PREMs for quality improvement?

o Yes

o No

Q17 Do you or your team use PREMs for program evaluation?

o Yes

o No

Q26 Do you use PREMs or PROMs for any purposes not listed above?

Q18 If you use PROMs and/or PREMs, how do you administer and collect the data? Please select all that apply.

▢ via mail

▢ by phone

▢ electronically, via email

▢ electronically at point of care

▢ Other (please explain)

Q19 If you use PROMs and/or PREMs, how do you report the data?

▢ At Individual Level

▢ At Aggregate Level

▢ N/A

Q20 If you do not currently use PREMs and/or PROMs, would you be interested in using them?

o Yes

o No

Q28 If interested in using PREMS and/or PROMS, please describe what you would like to measure or how PREMs/PROMs could be used in your practice:

Q22 Do you use these PROMs and/or PREMs in the area of child or youth (ages 0-24) mental health?

o Yes (if yes, describe which ones you use specifically for mental health)

o No

Q24**:** Thank you for taking the time to complete this survey. If possible, we would like to learn more from you in a future phone or Zoom call (no longer than 30 minutes). Would you be willing to chat further with us?

o Yes

o No

Q25

Please complete this Google form so that the researchers can schedule an interview: https://forms.gle/YUxTFVagzP6e4YFX8

Your name and contact information will NOT be associated with your responses on this survey.

**APPENDIX 2:** The interview guide to collect qualitative data on the use of pediatric PROMs and PREMs in Alberta and the challenges associated with their implementation

**Interview Guide**

1. How do you define PROMs/PREMs?
2. Have you ever heard about the use of prems/patient reported outcome measures in routine clinical care?
3. In your opinion, what skills are needed to use patient reported outcome measures/patient experience measures? (prompt –are there any other skills that you need?)
4. How confident are you that patients will receive good care through the use of PROMs/PREMs?
5. What do you think are the benefits of incorporating PROMs/PREMs in routine clinical care?
6. Are there any incentives for you to incorporate PROMs/PREMs in routine clinical care? If yes, what are they?
7. On a scale of 1-10 and 10 being very important, how important do you think it is for you personally to incorporate PROMs/PREMs in routine clinical care?

**If a participant uses PROMs/PREMs:**

1. How do you use PROMs/PREMs in your organization?
2. How are they administered in your clinic/organization?
3. Have there been any barriers to implementing PROMs/PREMs?
4. What has been your experience with using PROMs/PREMs?
5. Why do you use PROMs/PREMs?
6. What resources/support is required for you to continue using PROMs/PREMs?

**If the participant does not use PROMS/PREMS**

1. Would you be interested in using PROMs/PREMs?
2. Are there any current barriers to using PROMs/PREMs?
3. What resources/support are required for you to use PROMs/PREMs
